# Supplementary material for: YAP and ECM Stiffness: Key Drivers of Adipocyte Differentiation and Lipid Accumulation
Source: Cells. 2024 Nov 18;13(22):1905. doi: 10.3390/cells13221905 (PMC11593301; doi:10.3390/cells13221905)
Supplement: Supplementary file 1 [file cells-13-01905-s001.zip › cells-3268182-supplementary.pdf]

Supplementary Data Figures:

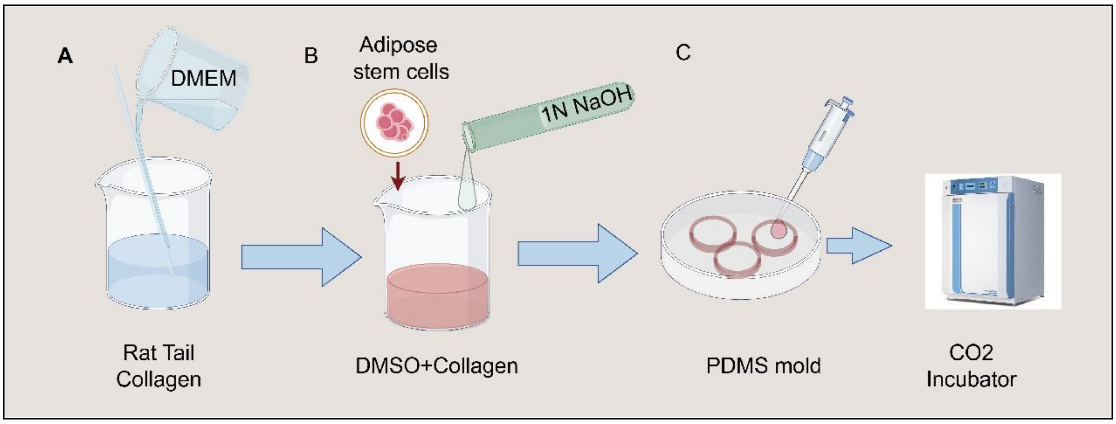

Supplementary Figure S1. Schematic Diagram of Hydrogel Preparation Process.

Figure A: Collagen from rat tail at a concentration of 3.47 mg/mL is mixed with different concentrations of DMEM to adjust the hydrogel concentration. **Figure B:** NaOH is added according to the collagen concentration to adjust the pH to 7.2, followed by the addition of the cell suspension. **Figure C:** The mixed hydrogel is injected into pre-prepared PDMS molds and incubated at 37°C to complete gelation.

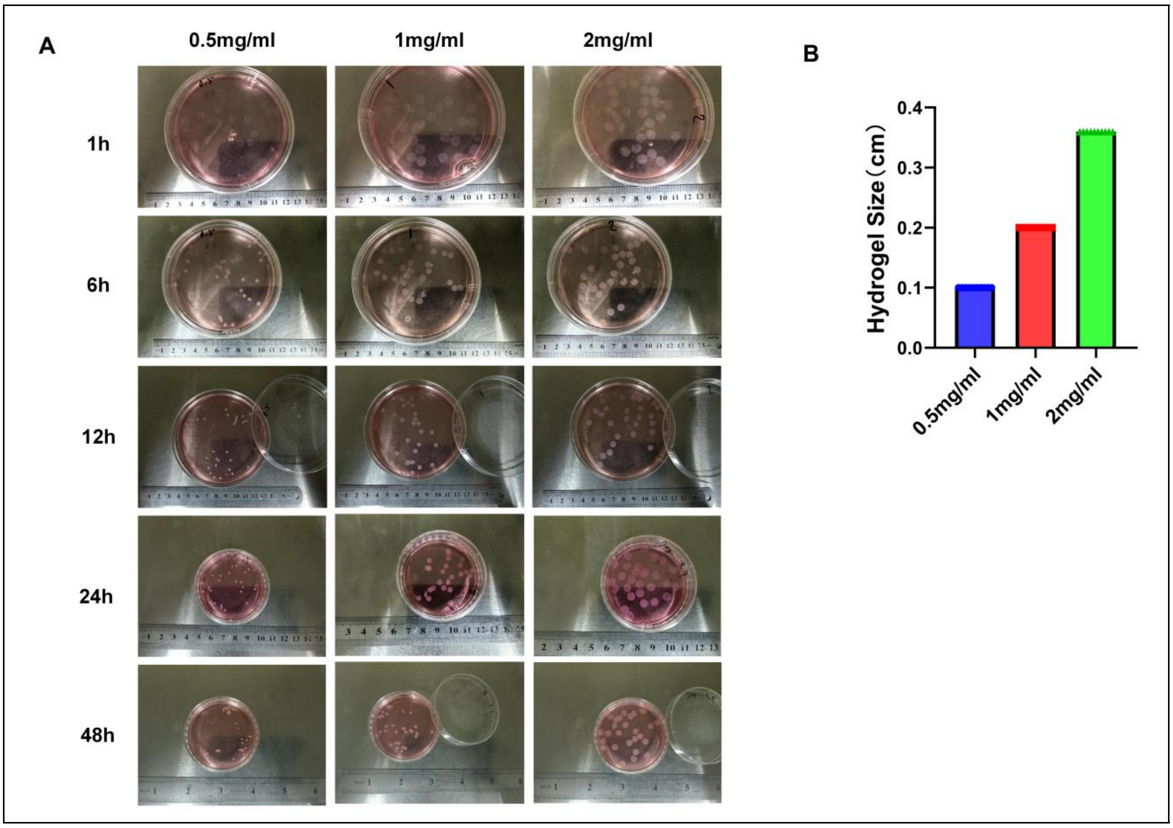

Supplementary Figure S2. Hydrogel Contraction Test at Different Time Points.

Figure A: Images of hydrogels taken at different time points, with a ruler used as a reference scale. **Figure B:** The degree of hydrogel contraction is measured using the ruler as a standard. Due to inconsistent camera positioning during imaging, apparent differences in hydrogel size were observed. Therefore, the final contraction measurements were standardized using the ruler as the reference.

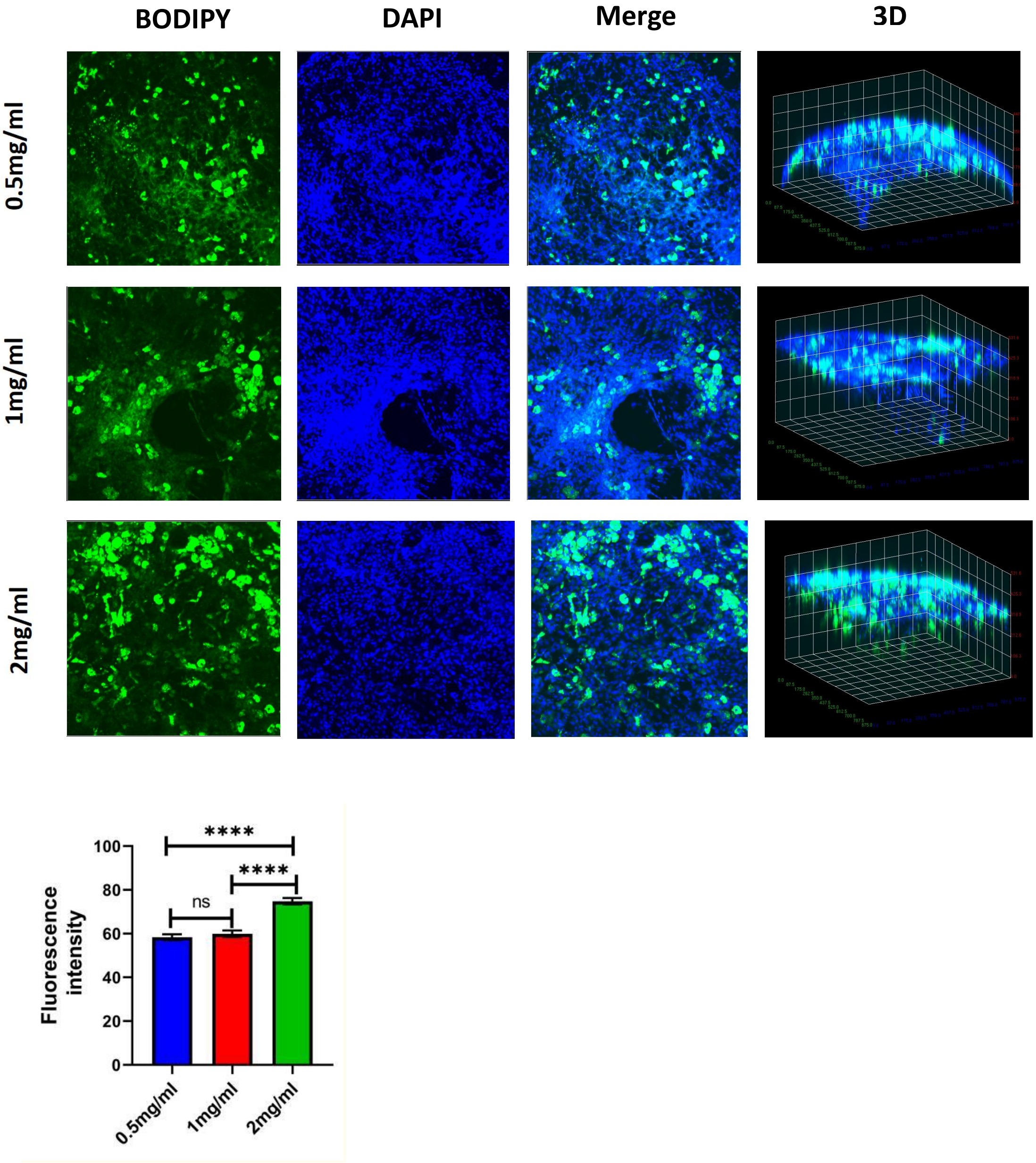

Supplementary Figure S3: 3D Distribution of Lipid Droplets in Hydrogels Observed by Laser Scanning Confocal Microscopy.

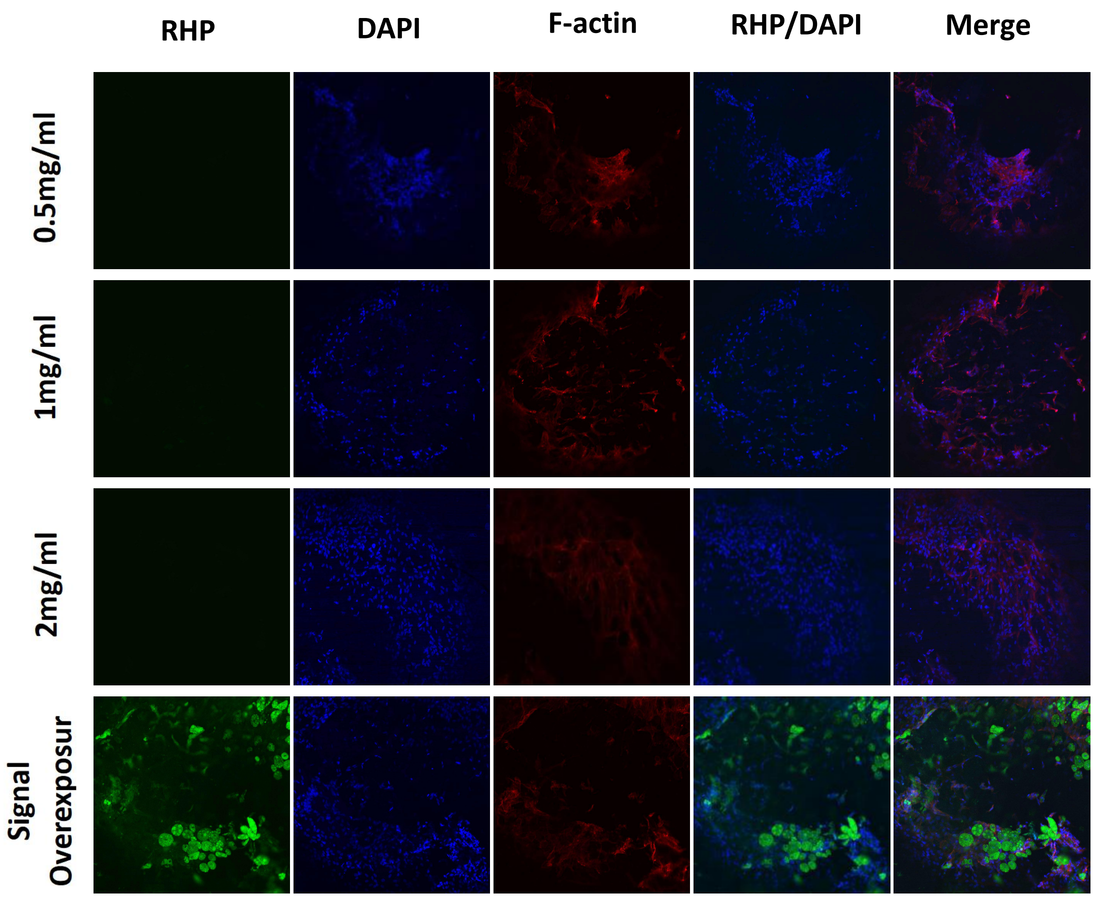

Supplementary Figure S4: YAP Immunofluorescence Negative Control.

The negative control for YAP immunofluorescence was conducted similarly to the standard immunofluorescence protocol, with the exception that the primary antibody (YAP) was not incubated. Only the secondary antibody (mouse anti-Rabbit HRP) was used. In the results, we did not observe strong spontaneous fluorescence from collagen. This indicates that collagen does not produce green fluorescence under normal exposure conditions. However, under overexposed conditions, a green fluorescence signal similar to that inside lipid droplets can appear. Previous studies have reported that lipid droplets exhibit specific autofluorescence around 488 nm.
